# Evanescent Wave Fiber Optic Biosensor for *Salmonella* Detection in Food

**DOI:** 10.3390/s90705810

**Published:** 2009-07-21

**Authors:** Angela M. Valadez, Carlos A. Lana, Shu-I Tu, Mark T. Morgan, Arun K. Bhunia

**Affiliations:** 1 Molecular Food Microbiology Laboratory, Department of Food Science, Purdue University, 745 Agriculture Mall Drive, West Lafayette, Indiana 47907, USA; E-Mails: valadeza@gmail.com (A.M.V); mmorgan@purdue.edu (M.T.M); 2 School of Aeronautics & Astronautics, Purdue University, 701 W. Stadium Ave., West Lafayette, Indiana 47907, USA; E-Mail: lanacarlos@yahoo.com.ar (C.A.L.); 3 United States Department of Agriculture, Agricultural Research Service, Eastern Regional Research Center, 600 E. Mermaid Lane, Wyndmoor, Pennsylvania 19038, USA; E-Mail: ShuI.Tu@ars.usda.gov (S.I.T.)

**Keywords:** *Salmonella*, fiber optic sensor, time–resolved fluorescence assay, egg, chicken

## Abstract

*Salmonella enterica* is a major food-borne pathogen of world-wide concern. Sensitive and rapid detection methods to assess product safety before retail distribution are highly desirable. Since *Salmonella* is most commonly associated with poultry products, an evanescent wave fiber-optic assay was developed to detect *Salmonella* in shell egg and chicken breast and data were compared with a time-resolved fluorescence (TRF) assay. Anti-*Salmonella* polyclonal antibody was immobilized onto the surface of an optical fiber using biotin-avidin interactions to capture *Salmonella*. Alexa Fluor 647-conjugated antibody (MAb 2F-11) was used as the reporter. Detection occurred when an evanescent wave from a laser (635 nm) excited the Alexa Fluor and the fluorescence was measured by a laser-spectrofluorometer at 710 nm. The biosensor was specific for *Salmonella* and the limit of detection was established to be 10^3^ cfu/mL in pure culture and 10^4^ cfu/mL with egg and chicken breast samples when spiked with 10^2^ cfu/mL after 2–6 h of enrichment. The results indicate that the performance of the fiber-optic sensor is comparable to TRF, and can be completed in less than 8 h, providing an alternative to the current detection methods.

## 1. Introduction

*Salmonella enterica* is one of the major food-borne pathogens of concern in the United States and other countries. Meat and poultry are considered the traditional sources; however, in recent years, fruits and vegetables [1], almonds [2] and peanut butter [3] have emerged as nontraditional vehicles. It is estimated that some 1.4 million *Salmonella* infections occur each year in the United States, with more than 500 deaths annually, leading to an annual cost of more than two billion dollars [4,5]. Between 1975 and 1987, the proportion of all *Salmonella* isolates represented by *S. enterica* serovar Enteritidis (*S*. Enteritidis) doubled in the United States [6,7], tripled in several European countries and increased by a factor of 275 in Argentina [8].

Improved hygienic practices have reduced but not eliminated the presence of this microorganism in the food supply. Antibiotic treatment of infection has been successful in the past; however, this has lead to the emergence of new multi-drug resistant *Salmonella* strains like *S. enterica* serovar Typhimurium DT104 [9]. There are also substantial costs associated with *Salmonella* infection resulting not only from medical expenses, but also from product recalls and company bankruptcies.

Presently, accurate and rapid pathogen testing methods are considered essential by the food industry. Typical detection methods of *Salmonella* using certified protocols outlined by the United States Department of Agriculture (USDA) or the Food and Drug Administration (FDA) take five to seven days for completion. These include the following basic steps: pre-enrichment, selective enrichment, isolation of pure culture, biochemical screening and serological confirmation. Due to the time required for standard methods, there is a need to develop rapid, sensitive, and specific detection tools for *S. enterica* serovar Enteritidis.

Over the past decade, biosensor technology has been intensively studied as a sensitive and reliable detection tool that is rapid enough for near real-time detection of microorganisms [10,11]. Biosensors enable researchers to obtain data instantaneously for detection of a specific analyte in a minimum amount of time and preparation steps, compared to conventional methods [12]. They can also allow for the detection of a broad spectrum of analytes in complex sample matrices (food, blood, urine and serum) [10].

The use of optical biosensors is one way to reduce the amount of time to detect pathogens in the food supply [13]. Fiber-optic biosensors are one of the most widely studied for rapid detection of many pathogens, toxins, proteins, and hormones [14–16]. A typical fiber-optic sensor operates by using a laser-diode to generate an evanescent wave along an optical waveguide to activate a fluorophore for detection. The basic principle is to link a specific polyclonal or monoclonal antibody, that binds to a target analyte, to the core of the fiber-optic waveguide. The evanescent wave excites any secondary antibody that is both conjugated with fluorescence molecules and bound with an antigen, such as bacteria. A portion of the resulting fluorescence light travels back through the waveguide and is measured using a photosensor [16]. The fluorescence is proportional to the amount of antigen or hapten present in the sample. One such sensor based on this immunosensing concept is the Analyte 2,000 (Research International, Monroe, WA, USA) [17–19].

Briefly, the Analyte 2,000 utilizes a laser (635 nm) to excite the flourophore and a photodiode to measure the fluorescence light. The system is capable of monitoring four optical fiber probes (biosensors) simultaneously using evanescent wave immunosensing. The output of each biosensor is an electric current proportional to the power of the fluorescence light measured by a photodiode. A computer interface allows for recording of the signals during use.

A fiber-optic biosensor has been used to detect *S*. Typhimurium at a limit of detection (LOD) of 50 cfu/g in spent irrigation water used in the sprouting of seeds [20]. In addition, a novel fluorescence resonance energy transfer (FRET)-based optical fiber biosensor was used for rapid detection of *S*. Typhimurium from homogenized pork samples with a calculated LOD of 10^3^ cfu/mL [21]. *E. coli* O157:H7 has also been detected using fiber-optic system (Research International, Inc.) at a concentration of 3–30 cfu/mL and 10^3^ cfu/mL in ground beef [22,23]. Finally, *Listeria monocytogenes* was detected at a concentration of 10^3^–10^4^ cfu/mL in hotdog [24,25]. Although this technology has been used previously to detect *Salmonella* Typhimurium [20], method for *S*. Enteritidis is not available and it is the second most commonly occurring serovar [6,7].

The goal of this project is to employ fiber optic sensor for detection of *S*. Enteritidis from spiked food samples in less than 8 h beginning with the food sample and compare the results with previously developed time-resolved fluorescence (TRF) assay. Using the four-channel Analyte 2,000 (Research International, Inc.), we employed Alexa-Fluor 647 conjugated antibody to detect *S*. Enteritidis initially from buffer and then from spiked shell eggs and chicken breast only after 2–6 h of enrichment so that the assay could be completed within an 8-h shift in a processing facility. We also verified the specificity of the fiber optic sensor by testing with common foodborne microflora likely to be encountered in food products during testing. Furthermore, we compared the fiber-optic sensor to a sensitive TRF assay for confirmation [26], which has been developed for detection of *S.* Typhimurium, *E. coli* O157:H7 and biological warfare agents, including *Francisella tularensis*, *Clostridium botulinum* neurotoxin and *Staphylococcus aureus* enterotoxin [27–30].

## 2. Results and Discussion

### 2.1. Reaction Characteristics of Anti-*Salmonella* PAb and Anti-*Salmonella* MAb 2F-11 by ELISA

*S*. Enteritidis Phage Types Showed a Stronger Reaction Compared to the Other Tested Bacterial species in the indirect Enzyme Linked Immunosorbent Assay (ELISA) (data not shown). This reaction profile analysis showed PAb and MAb 2F-11 reacted with all phage types of *S*. Enteritidis and was detectable above 10^6^ cfu/mL. PAb generally had higher reactivity compared to MAb 2F-11, mainly due to the presence of higher numbers of binding epitope sites on *S*. Enteritidis. When PAb concentration at 0.49 μg/mL was examined, *Staphylococcus aureus* and *E. coli* O157:H7 showed very strong reactions (>3.0 absorbance at 490 nm); equivalent to *S*. Enteritidis PT4. *Proteus vulgaris* and *Listeria monocytogenes* showed moderate reactions (Abs_490nm_ = 0.7–1.1) and *Lactobacillus rhamnosus*, *Enterococcus faecalis, Lactobacillus gasseri*, and *Pseudomonas aeruginosa* showed weak to poor reactions. The reason for increased reaction with *S. aureus* can be attributed to the production of Protein A which binds the Fc part of IgG subclass antibodies. *E. coli* O157:H7 and *P. vulgaris* possibly share antigenic properties with *Salmonella* thus showing high reactivity with PAb [31].

In the case of MAb 2F-11, overall, it showed strong reactions with *S*. Enteritidis PT4 and *S. aureus,* and exhibited weak cross-reactions with remainder of tested cultures. The best differential reaction was seen at antibody concentration of 3.82 μg/mL. As indicated above increased reaction with *S. aureus* is due to its protein A-mediated reaction with antibody. *S*. Enteritidis reactivity decreased greatly above the concentration of 1.91 μg/mL.

### 2.2. Sensitivity and Specificity of Fiber-Optic Biosensor

**Figure 1.**
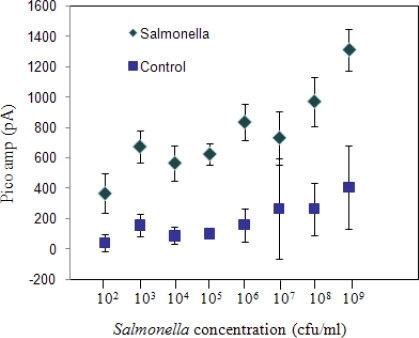
Sensitivity of the immunosensor using serial dilutions of *S*. Enteritidis cells suspended in phosphate buffered saline (PBS). Controls are devoid of bacteria.

A limit of detection test was performed to evaluate low estimated concentrations of pure cultures of *S*. Enteritidis in sterile phosphate buffered saline (PBS) detectable with the fiber-optic system (Figure 1). *S*. Enteritidis concentration of 1 × 10^9^ cfu/mL gave the strongest signal, (1,047 ± 92 pA) and in general, the signal decreased with a decrease in bacterial concentration. The lowest cell concentration that gave a positive signal in comparison to the background control (no bacteria) was 1 × 10^3^ cfu/mL (717.14 ± 53.6 pA) and was considered to be the detection limit for the sensor. “Limit of detection” was considered as the minimum concentration that was significantly greater than (P < 0.05) the signal from the negative control (158.5 ± 105.8 pA). One-way ANOVA analysis indicated that the readings from control experiments performed for each experiment (P < 0.05) were not different. In the case of the concentration readings, one-way ANOVA with concentration as the variable indicated that the readings were significantly affected by the bacterial concentrations (P < 0.001).

Evaluation of the sensor with other bacterial species was compared independently with the positive control (*S*. Enteritidis) each at a concentration of ∼5 × 10^8^ cfu/mL (Table 1). Emission values ranged from 170–713 pA for each bacterial species tested. The control (without bacterial culture) values ranged from 207–453 pA. Positive result was assigned based on Tukey’s grouping at P < 0.05 and at least 2X of background controls (fiber without bacteria). The fiber optic sensor gave positive results with *S*. Enteritidis and *S*. Typhimurium, and negative results with *Enterococcus faecalis*, *Escherichia coli* O157:H7, *Lactobacillus rhamnosus* and *Listeria monocytogenes*. Positive result with *S. aureus* was expected since this bacterium expresses Protein A that binds to IgG (Table 1).

**Figure 2.**
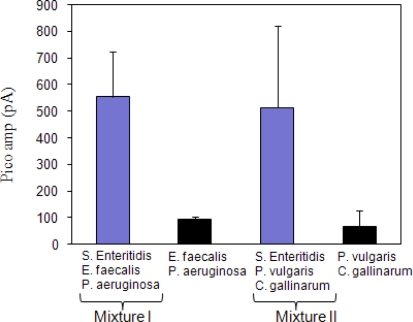
Fiber-optic signal recognition of *Salmonella* Enteritidis in a mixed bacterial culture. In mixture I, *S.* Enteritidis was used at 1 × 10^5^ cfu/mL while *Enterococcus faecalis* and *Pseudomonas aeruginosa* at 1 × 10^6^ cfu/mL each. In mixture II, *S.* Enteritidis was used at 1 × 10^5^ cfu/mL while *Proteus vulgaris* and *Carnobacterium gallinarum* at 1 × 10^6^ cfu/mL each.

In the case of the mixed culture competition test, two mixed cultures were tested to evaluate if *S*. Enteritidis was detectable in the presence of other microorganisms (Figure 2). In a mixed culture experiment I, (*S*. Enteritidis, *E. faecalis* and *P. aeruginosa*) fiber optic signal was in 550 pA range which was significantly different (P = 0.0105) from mixture without *S*. Enteritidis. Similar results were also seen in mixed culture experiment II (*S*. Enteritidis, *P. vulgaris* and *C. gallinarum*) (P = 0.0029). The data indicate that the sensor was able to detect *Salmonella* in the presence of other bacterial cultures.

Overall, the fiber-optic sensor has a detection limit for pure *S*. Enteritidis cells at 10^3^ cfu/mL. Other bacterial species exhibited negative results compared to *Salmonella* in the specificity test. When a low concentration of *S*. Enteritidis (10^5^ cfu/mL) was mixed with a high concentration of commensal microflora (ea. 10^6^ cfu/mL), the sensitivity of the data from the sensor was not compromised. This observation implies that the presence of natural microflora may not interfere with the binding of antibodies to *S*. Enteritidis, thus affecting the sensitivity or selectivity of detection. Previous results showed similar LOD testing in other bacterial species compared to this study. For *S*. Typhimurium, there was a LOD of 50 cfu/g in spent irrigation water used in the sprouting of seeds [20] while a novel FRET-based optical fiber biosensor was used for rapid detection of *S.* Typhimurium cells in pork samples with a detection limit of 10^3^ cfu/mL [21]. As in the case of *E. coli* O157:H7, it was detected at a concentration of 3–30 cfu/mL and 10^3^ cfu/mL in ground beef [22,23]. Finally, *L. monocytogenes* was detected at a concentration of 10^3^–10^4^ cfu/mL in hotdog and bologna samples [24,25].

### 2.3. Sensitivity and Specificity of Time-Resolved Immunofluorescence (TRF) Assay

**Figure 3.**
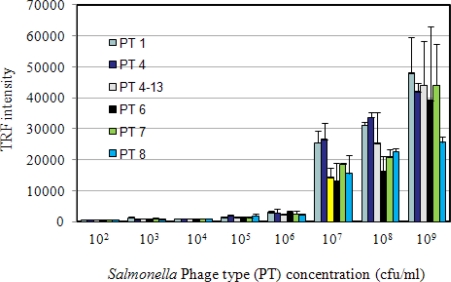
Sensitivity of immunomagnetic bead time-resolved immunofluorescence (IMB-TRF) for detection of *Salmonella* Enteritidis phage types (PT).

In this study, we employed immunomagnetic bead time-resolved immunofluorescence (IMB-TRF) in a sandwich configuration to compare results with the fiber-optic biosensor. Pure cultures of *S.* Enteritidis in sterile PBS were used to determine the limit of detection, and specificity was determined by testing with other bacterial species and spiked food samples. TRF reactions with different concentrations of *S*. Enteritidis phage types (PT1, PT4, PT4–13, PT6, PT7 and PT8) are presented in Figure 3. The highest TRF values for all PT’s was around 50,000 and the variations among phage types were minimal. The detection limit was calculated to be 10^3^ cfu/mL based on 2X the control values (673.0), as was done in previous studies [27,32]. ANOVA analysis compared TRF intensity values at each bacterial concentration, and values were significantly different from each other (P < 0.0001), which indicates TRF intensity was concentration dependent.

**Figure 4.**
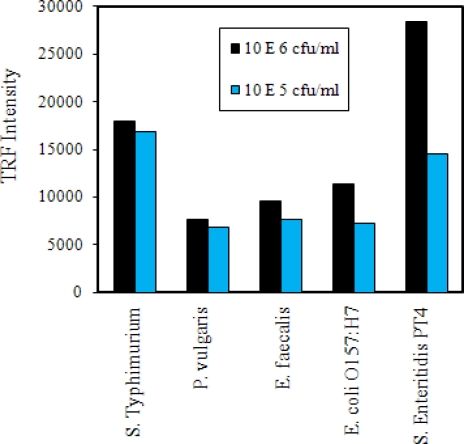
Selectivity tests of immunomagnetic bead time-resolved immunofluorescence (IMB-TRF). Values are from an average of two wells each tested with two different concentrations of cells.

A selectivity test was performed by testing other bacterial species at levels of 10^6^ and 10^5^ cfu/mL (Figure 4). As expected, the highest TRF reaction was seen with *S.* Enteritidis at 10^6^ cfu/mL. Similar reactions were also seen in *S*. Typhimurium at 10^6^ and 10^5^ cfu/mL levels. TRF reactions for other bacterial cultures at 10^6^ and 10^5^ cfu/mL levels were below the values seen for *Salmonella.* A positive reaction with *S*. Typhimurium is highly desirable since all *Salmonella* serovars are considered pathogenic [5] and this would allow detection of both serovar. ANOVA analysis indicated that the TRF values between *Salmonella* serovars and the other species tested were significantly different from each other (P = 0.0020).

Taken together, these results indicate that IMB-TRF method could be used for detection of low levels of pure cultures of *Salmonella* without any cross-reactions with other bacterial species tested. Previously IMB-TRF was used to detect *Salmonella* spp. and *E. coli* O157:H7 from different food samples such as ground beef, liquid eggs and alfalfa sprouts [27–29,33]. They were able to detect ∼10^4^ cfu/mL of various strains of *S*. Typhimurium and ∼10^2^ cfu/mL of *E. coli* O157:H7 after 4.5 h enrichment at 37 °C.

### 2.4. Detection of *S*. Enteritidis Grown in Shell Egg and chicken Breast Using Fiber Optic and TRF Assay

Food samples (egg and chicken breast) spiked with *S*. Enteritidis at 10^2^ cfu/mL were tested with fiber-optic and TRF sensors to validate the robustness and to evaluate any possible interferences from food matrices. Sensors were applied to the same samples that were collected every 2 h intervals during enrichment at 37 °C.

#### 2.4.1. Fiber-optic assay

**Figure 5.**
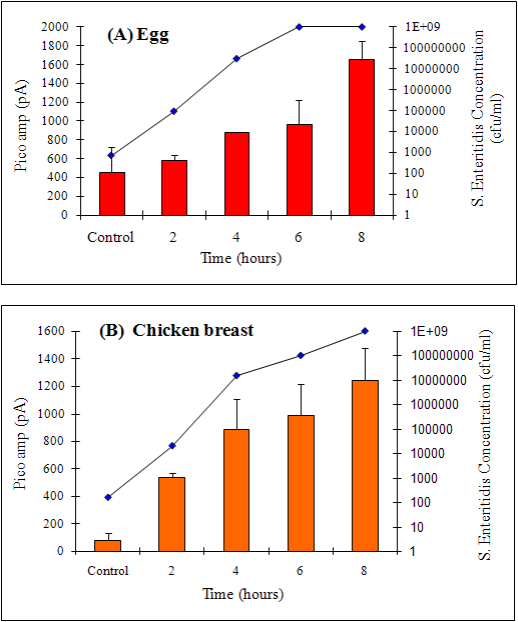
Detection of *S.* Enteritidis grown in egg (A) and chicken breast (B) at 2 h intervals by using the fiber-optic biosensor. Bars represent the signals (left Y axis) from the biosensor. Line (growth curve) represents concentrations (right Y axis) of *S*. Enteritidis grown in egg suspended in TSB (A) and chicken breast (25 g in 225 mL TSB) (B). A total of 5-eggs were used for this experiment, one egg was used for each time point.

For *Salmonella* cells grown in egg after 2 h of enrichment, counts reached to 9.45 × 10^4^ cfu/mL and the fiber-optic signal was 584.74 pA, (P = 0.0226) which was slightly higher than the control of 459.9 pA (Figure 5A). *S.* Enteritidis growth reached a plateau in 6 h at 1 × 10^9^ cfu/mL. These data indicate that *S.* Enteritidis is detectable in egg as early as 4 h with initial inoculation levels of 10^2^ cfu/mL. In chicken breast, *Salmonella* growth was relatively slower at 2 h with a bacterial count of 2 × 10^4^ cfu/mL and a corresponding signal of 539.68 pA (P = 0.0011) was obtained from the biosensor (Figure 5B). The signal increased as the enrichment times increased. Taken together, it was determined that the fiber-optic biosensor could detect *S*. Enteritidis positively in both egg and chicken after only 4 and 2 h of enrichment, respectively (ANOVA analysis, for egg; P = 0.0029 and chicken; P < 0.0001) when spiked with 10^2^ cfu/mL.

Previously, Geng *et al.* [23] was able to detect *E. coli* O157:H7 using the fiber-optic sensor only after 4 h of enrichment from ground beef with an initial inoculation of 1 cfu/mL. In this study, we were able to detect *S*. Enteritidis at an inoculation level of ∼10^2^ cfu/mL in both egg and chicken breast suspended in TSB after 4 and 2 h of enrichment, respectively. Fiber-optic sensors have been applied for detection of *Salmonella* from various food sources with variable detection limits as mentioned previously.

#### 2.4.2. TRF assay

**Figure 6.**
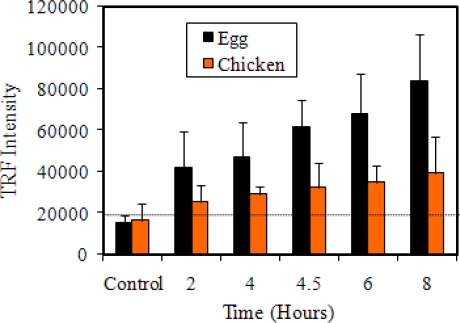
Detection of *Salmonella* Enteritidis spiked in egg and chicken by IMB-TRF. A total of 24 eggs were used for this experiment, four eggs for each time point.

TRF result with *S*. Enteritidis spiked in both egg and chicken breast is presented in Figure 6. At 2 h, TRF values for egg sample were in the 40,000 range and increased proportionally as enrichment time increased, compared to the control. During the same enrichment period with chicken breast sample, the TRF values were in the 30,000 range, which slightly increased as the enrichment time increased. These data indicate that *S*. Enteritidis could be detected in egg after 2 h of enrichment at 37 °C with a TRF intensity of 41,896 ± 17,967 and a concentration of 9.4 × 10^4^ cfu/mL compared with the average control of 15,388 ± 3,378 (P = 0.0002).

For chicken breast, it took 6 h for a positive result. The TRF intensity was 35,026 ± 7,944 for *Salmonella* counts of 1.0 × 10^8^ cfu/mL, which was numerically higher than the control of 16,665 ± 7,824 but lacked statistical significance (P = 0.1608). The reason for the lower TRF intensity of chicken breast compared to egg could be due to the presence of competing microflora, microbial enzymes and other natural inhibitors in raw chicken breast, which probably affected the TRF assay. Using TRF assay, Tu *et al.* [33] detected *Salmonella* Typhimurium from liquid egg after 20 h enrichment with initial inoculation of 1 cfu/egg and after 5 h enrichment, with an initial inoculation of 10 cfu/egg.

Comparison of both fiber-optic and TRF methods for detection of *Salmonella* from spiked food samples revealed that fiber-optic assay is as sensitive as TRF since both methods allowed detection as early as 2 h of enrichment. Total assay time beginning with the enriched food sample for fiber-optic was 1.5 h and for TRF was 2.5 h.

## 3. Experimental Section

### 3.1. Cultures and Media

*Salmonella* Enteritidis phage types (PT) 1, 4, 4–13, 6, 7, and 8 from our culture collection were maintained on brain heart infusion (BHI; Difco Lab) agar (1.5%) plates at 4 °C. For use with fiber-optic assay, *S*. Enteritidis was grown in BHI broth for 17 h in a 37 °C shaking incubator and serially diluted in sterile 0.02 M phosphate buffered saline (PBS; pH 7.4). For selective enrichment and enumeration, *S*. Enteritidis cells were cultured on xylose lysine deoxycholate (XLD) or xylose lysine tergitol 4 (XLT-4) selective agar plates (Difco). *Salmonella* Typhimurium, *Escherichia coli* O157:H7, *Listeria monocytogenes* V7, *Enterococcus faecalis* CG110, *Pseudomonas aeruginosa* ATCC10145, *Proteus vulgaris*, *Lactobacillus rhamnosus*, *Lactobacillus gasseri*, *Carnobacterium gallinarum* ATCC49517, and *Staphylococcus aureus,* were also maintained on BHI agar plates at 4 °C for the duration of the study. All fresh cultures for experiments were obtained by inoculating a loop of colonial cultures into BHI broth and incubating them at 37 °C for 17 h with shaking. *Lactobacillus* cultures were grown in deMann Rogosa Sharpe (MRS) broth and fresh samples were grown at 37 °C for 17 h with 7% CO_2._

### 3.2. Antibodies and Labeling

The anti-*Salmonella* rabbit polyclonal antibody (PAb) was produced against heat-inactivated *S*. Enteritidis cells in our laboratory (unpublished) and used as capture antibody. The PAb (2 mg/mL) was purified by using Hitrap™ Protein-A purification system (Amersham Biosciences, Piscataway, NJ) and labeled with a long-chain biotin (EZ-Link NHS-LC-Biotin; Pierce, Rockford, IL) according to the manufacturer’s instruction. The final biotinylated antibody concentration was estimated to be 2.0 mg/mL and stored in PBS with 0.1 % thimersol at 4°C until used.

Anti-*Salmonella* 2F-11 monoclonal antibody specific for lipopolysaccharide (LPS) of *Salmonella* Enteritidis was used as a reporter [34,35]. Purified MAb 2F-11 (1.53 mg/mL) was labeled with Alexa Fluor 647 (Invitrogen, Carlsbad, CA) according to manufacturer’s instructions. The final concentration of Alexa Fluor 647-labeled antibody (AF-MAb) was 2.2 mg/mL, and the final molecular ratio of dye to antibody was estimated to be 1 mole dye per mole of protein. For TRF assay, MAb 2F-11 was labeled with samarium (Sm^3+^) using DELFIA^®^ Sm-labeling kit (Perkin-Elmer, Wellesley, MA). The calculated yield of (Sm^3+^/IgG) was 0.51.

### 3.3. Fiber Preparation, Blocking and Background Reading

The polystyrene waveguides (4 cm in length and 0.78 mm in diameter: Research International), were pre-cleaned with 100% isopropanol followed by deionized water using a sonicator, and air-dried. The fibers were rinsed with PBST (PBS containing 0.5% Tween 20) before experiment initiation [24].

A flow-through system was used for immobilization of anti-*Salmonella* PAb onto the polystyrene fibers [25]. The system consisted of 4 channels (waveguide holders) where three were used for sample testing while one for control. The waveguide holders were connected to a peristaltic pump (Ismatec, Wertheim-Mondfeld, Germany). Fibers were first incubated with 100 μg/mL of streptavidin (Sigma) overnight for 16–20 h. The fibers were then rinsed with PBST and then incubated with biotinylated anti-*Salmonella* PAb (0.05 mg/mL suspended in PBS containing 2 mg/mL of BSA) at room temperature for 1.5 h. Fibers were rinsed again with PBST and then incubated with Superblock (Pierce, Rockford, IL, USA) for 1 h followed by reaction with biotinylated bovine serum albumin (B-BSA; 1 mg/mL) (Pierce) for 30 min to block nonspecific binding sites. A background reading was taken after a final wash with PBST. This reading value, recorded in Pico amperes (pA), was considered to be the background for each fiber from each channel. All four channels were connected in parallel with Pharmed tubing with an inner diameter of 0.88 mm (Bio-Rad, Hercules, CA, USA). All fluids were pumped at 500 μL per min at room temperature.

### 3.4. Fiber-optic Assays

A volume of 3 mL of bacterial cells suspended in sterile PBS, pH 7.4, was injected into the pump system and incubated with fibers at room temperature for 1 h. After rinsing with PBST, AF-MAb 2F-11 was injected into the system and incubated for 15 min and a reading was taken. Then the fiber was rinsed with PBST for 6 min, and a final reading was taken for 90 sec and values were subtracted from the background readings from fibers exposed to PBS only. For each concentration of *S*. Enteritidis, or each of the bacterial species, three to six fibers were used to generate average values and standard deviations.

### 3.5. Sensitivity and Selectivity of Fiber-Optic Sensor

To determine the sensitivity (detection limit) of this sensor, fresh culture of *S*. Enteritidis was washed and serially diluted from 1 × 10^9^ to 1 × 10^2^ cfu/mL in sterile PBS. The specificity of the biosensor to discriminate *S*. Enteritidis from other microflora (*P. aeruginosa, C. gallinarum* ATCC 49517, *E. faecalis*, and *L. rhamnosus*) was tested. Concentration of each freshly grown culture was adjusted to uniform concentration and 3 mL of each was tested separately. To determine the fiber-optic signal response for *S*. Enteritidis cells in the presence of other bacteria, two mixed-culture conditions were used. In mixture I, *S*. Enteritidis (10^5^ cfu/mL) was mixed with 10^6^ cfu/mL of each *P. vulgaris* and *C. gallinarum*) and in Mixture II *S*. Enteritidis (10^5^ cfu/mL) was mixed with 10^6^ cfu/mL of each *E. faecalis* and *P. aeruginosa*. The controls consisted of mixed cultures without *S*. Enteritidis.

### 3.6. TRF Assays

The biotinylated antibody-linked streptavidin coated magnetic beads (IMB) diluted (100 μL/well) in assay buffer (Tris-HCL buffer, pH 7.8, solution with bovine serum albumin, gamma-globulin, Tween 40, diethylenetria, inepentaacetic acid (DTPA), an inert red dye and <0.1 % sodium azide as a preservative) (Perkin-Elmer, Wellesley, MA, USA) were incubated with the bacterial sample (100 μL/well) for 1 h using the King Fisher (Thermo Electron Corporation, Waltham, WA, USA). This system is an automated, programmable magnet manipulator that was used to concentrate, wash and label the sample to avoid error in sample handling [29]. Followed by a wash (200 μL/well) with washing buffer (Tris-HCL buffer, pH 7.8 solution containing Tween 20%) (Perkin-Elmer), 100 μL of the samarium (Sm)-labeled antibody was mixed with the bacteria-bead complex for 1 h and washed twice before the sample was added to enhancement solution (Triton X-100, acetic acid and chelators) so the Sm ions in the antibody-IMB complexes could be extracted (Perkin-Elmer). The readings were taken using the Wallac Victor 2 multilabel counter (Perkin-Elmer).

### 3.7. Detection of *S*. Enteritidis from Artificially Inoculated Shell Eggs and Chicken Breast

For each experiment, freshly prepared *S*. Enteritidis culture was aseptically transferred to 100 mL of TSB broth containing one large, USDA grade A egg or 25 g of raw chicken breast in stomacher bags with filters (Nasco, Fort Atkinson, WI, USA), separately. The food samples were purchased from a local grocery store in Philadelphia, PA. The samples were then homogenized for 1–2 min in a Seward Stomacher (Seward, England) and incubated at 37 °C in a shaker incubator for 8 h with constant shaking at 130 rpm. The final inoculum levels for each enrichment broth containing egg and meat samples were determined to be ∼10^2^ cfu/mL. The bacteria concentrations were determined using XLT-4 agar plates at 0, 2, 4, 6, and 8 h. Simultaneously, TRF and fiber-optic biosensors were applied to each sample and signals were obtained at 2 h intervals from 0 h to 8 h. In total, 24 eggs and 600 g of chicken breast were tested for the duplicates and trials. For the TRF assay, 100 μL; aliquots of sample was added to each well, in duplicates, in the 96-well plate and assayed as described above. For the fiber-optic sensor, 3 mL of sample was taken and analyzed as described above.

### 3.8. Statistical Analysis

Fiber-optic and TRF data were analyzed using the SAS program (Cary, NC) for ANOVA and the Tukey’s t test. Tukey’s multiple comparison tests was done to determine which mean amongst a set of means differed from the rest. ANOVA was done to evaluate differences between means of the populations. The P-value, mean and standard deviation were calculated. Differences in mean values were determined by Tukey’s test at P < 0.05.

## 4. Conclusions

The experimental results presented in this study will help to better define the reaction profiles of anti-*S*. Enteritidis PAb and anti-*S*. Enteritidis MAb 2F-11 for use in various immunosensor and biosensor applications. All phage types of *S*. Enteritidis tested showed positive reactions in ELISA, fiber-optic and IMB-TRF.

The data presented in this report suggest that the fiber-optic system is suitable for detection of *S*. Enteritidis from food samples. Previous studies using the fiber-optic sensor have either tested with one food sample or no food samples at all, and those did not validate the results using another contemporary tool. Other biosensors have shown some success with antibody binding to whole bacteria but higher concentrations and/or longer incubation times were needed to show positive results. The assay presented in this study is completed within an 8 h-work day and the results were validated using another popular, sensitive, detection system, TRF. Attempts to increase the sensitivity below 10^4^ cfu/mL in food were unsuccessful and could have been due to non-specific binding, which may require improved blocking reagents. Overall, the fiber-optic assay was able to detect *S*. Enteritidis from an initial inoculation of ∼10^2^ cfu/mL just within 2–6 h of enrichment in egg or chicken and was validated with the IMB-TRF system that required the equivalent enrichment times and bacterial concentrations.

## Figures and Tables

**Table 1.** Specificity of the fiber-optic sensor for *S*. Enteritidis when compared with other live microorganisms.

**Bacterial Culture**	**Avg (pA)^a^**	**Control (pA)**	**Results**
*Salmonella* Enteritidis	997.97 ± 91.85	285.08	Positive
*Salmonella* Typhimurium	868.13 ± 31.29	299.06	Positive
*Escherichia coli* O157:H7	570.00 ± 227.20	207.78	Negative
*Staphylococcus aureus*	906.39 ± 74.93	262.98	Positive
*Enterococcus faecalis*	789.87 ± 168.62	453.62	Negative
*Lactobacillus rhamnosus*	481.10 ± 166.96	311.07	Negative
*Listeria monocytogenes*	420.38 ± 95.86	270.60	Negative

a Values are average of six optical fibers from three independent experiments with bacterial concentration of 5 × 10^8^ cfu/mL and background (control) signal values, i.e., fibers exposed to buffer only are presented in separate column. Positives values were assigned based on Tukey’s grouping at P < 0.05.
